# Huge Facial Desmoid Tumors with Neck Extension: A Case Report

**Published:** 2014-07

**Authors:** Ali Ghazipour, Shervin Ghavami Lahiji, Bassel Bradd, Fariborz Saleheh

**Affiliations:** 1 *Department of Otorhinolaryngology, Jondishapour University of Medical Sciences, Ahwaz, Iran.*; 2 *Department of Otorhinolaryngology, Damascus University of Medical Sciences, Damascus, Syria.*; 3 *Department Oral and maxillofacial surgery, Damascus University of Medical Sciences, Damascus, Syria.*

**Keywords:** Fibromatosis, Fibrous Tissue, Mandibular Diseases, Neoplasms

## Abstract

**Introduction:**

Desmoid tumors are very rare, benign fibrous neoplasms arise from the musculoaponeurotic structures throughout the body.

**Case Report:**

The patient was a seven-year old boy with a large mandibular mass growing over a period of six months. His CT-scan showed a large mass, 13 cm in diameter in the cheek area extending to the neck and trachea. Biopsy was compatible with desmoid fibromatosis. He was given neoadjovant treatment with vinblastin and methotrexate. The patient underwent a tracheostomy. Then a complete hemimandibulectomy and submandibular gland excision was performed. Finally reconstruction with latisimus dorsi free flap was performed.

**Conclusion:**

Despite rarity desmoid tumors should be kept in mind of an otorhinolaryngologist as a differential diagnosis in children with head and neck mass.

## Introduction

Desmoid tumors are very rare, benign fibrous neoplasms arise from the musculoaponeurotic structures throughout the body. This overgrowth of fibrous tissue is typically infiltrative,well-defined, and firm (1). The overall incidence of desmoid tumors in childhood is estimated at 2–4 new diagnoses per 1 million per year and they are very uncommon in the head and neck especially in infants and young children (2,3). Only two cases of desmoid tumors of the cheek has been reported (4).Common anatomic sites for appearance of desmoid tumors include extremities, abdominal wall, and mesentery. Because this tumor is very rare (2–4 per 1 million) there are no controlled studies comparing the treatment options for this tumor but complete surgical excision of desmoid tumors is believed to be the only effective method of cure by most authors (1,5). Local recurrence rates after excision were reported to be as high as 70% (6). The pathogenesis of desmoid tumor is most likely multifactorial; genetic predisposition endocrine factors and trauma seem to play an important role (7-9).

The current paper presents a case of cheek desmoid tumor with poor response to chemotherapy.

## Case Report

**Fig 1:**
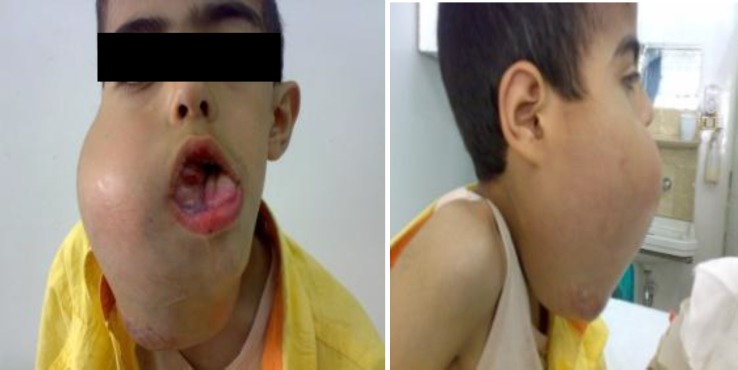
Clinical appearance

The patient was a seven-year old boy with a large mandibular mass growing over a period of six months. A biopsy was done few months before the patient came to our center and showed a myofibroblastic lesion compatible with desmoidfibromatosis. However, due to the size of the lesion and the location surgery could cause unacceptable morbidity and cosmetic and functional outcome;We decided to give him neoadjovant treatment. Chemotherapy with vinblastin and methotrexate was initiated, but after 4 weeks of treatment the tumor continued to grow causing local pain, dyspnea, odynophagia, dysphagia and inability to close the mouth. Physical examination showed a Very big hard mass extending from the right mandible to the mouth and neck (Fig.1). 

**Fig 2:**
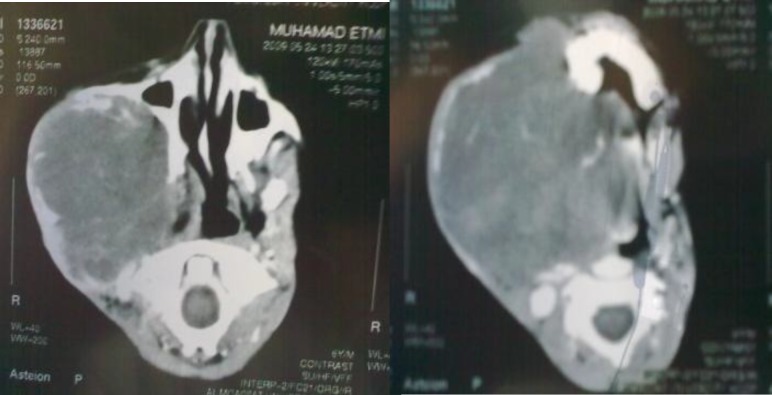
CT-scan of the patient

His CT-scan showed A large mass, 13 cm in diameter in the cheek area extending to the neck and trachea; enclosing and compressing upper third of it (Fig.2).

**Fig 3:**
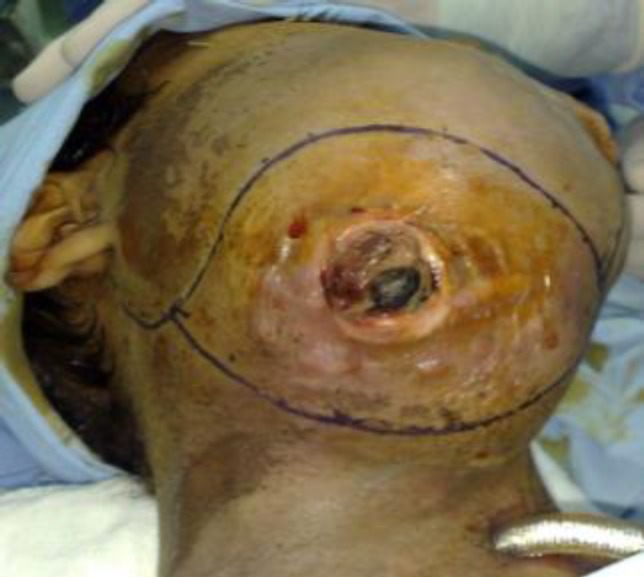
Photograph before surgery

**Fig 4:**
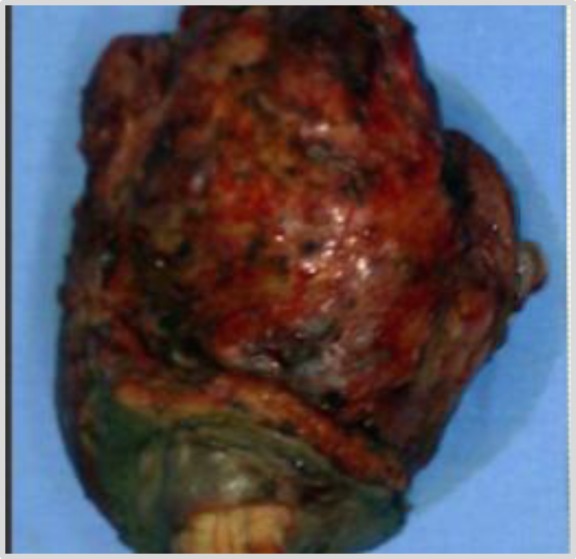
The excised mass

The patient underwent a tracheostomy in the ENT department and we decided to go for surgery. A complete hemimandibu- lectomy and submandibular gland excision was performed (Figs.3,4).

**Fig 5:**
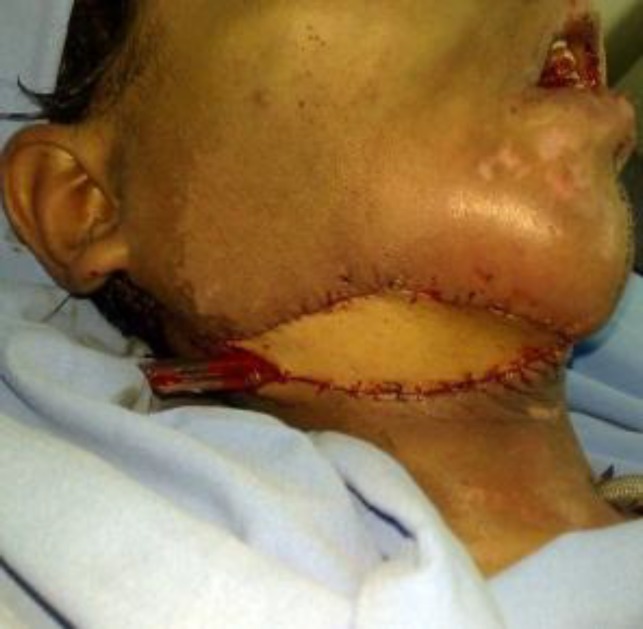
Outcome with latisimusdorsi free flap

**Fig 6:**
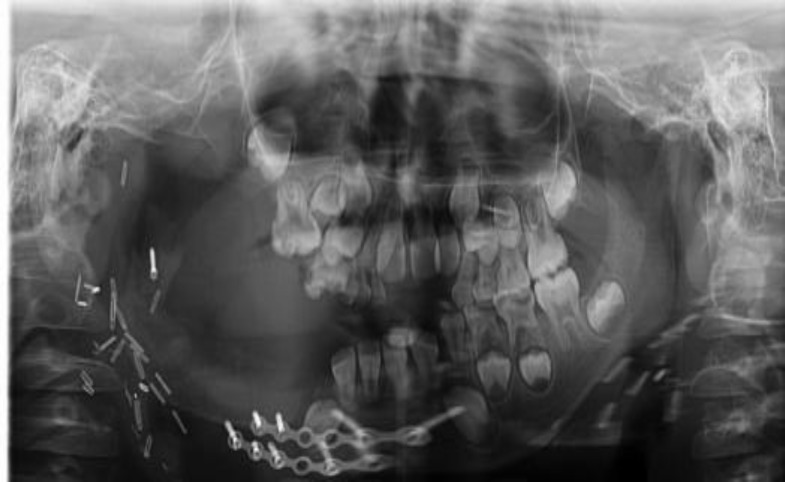
Reconstructed mandible and temporo- mandibular joint

**Fig 7:**
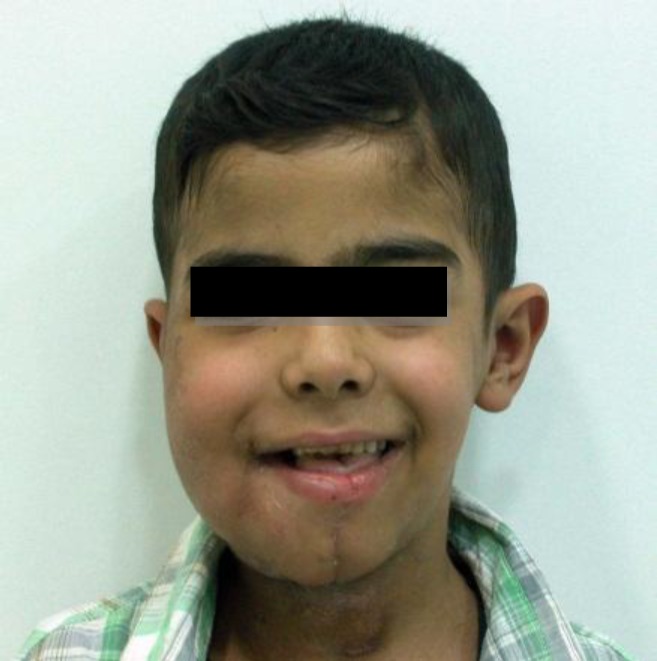
Out come after 1.5 years

Then a reconstruction with latisimusdorsi free flap was performed (Fig.5). Surgical margins were free. Six months later reconstruction of mandible and temporo- mandibular joint was performed by using iliac crest and costo-chondral rib graft (Fig6).Good cosmetic outcome and no recurrence were seen after 1.5 years of follow up (Fig.7).

## Discussion

Desmoid tumors are rare and account for 0.03% of all neoplasms (1) and only few cases of mandibular desmoids have been reported which suggest its exceptional location. These tumors are locally aggressive and nonmetastasizing. The cause of desmoids is not clear, but there is an association with genetics, local trauma and hormonally active states (7-9). Fewer than 15% of desmoids occur in the head and neck.

There are no specific clinical or radiographic findings. Histologic findings include spindle cells with rare mitoses and an abundant collagenous stroma with infiltration of the surrounding tissues.

Treatment of desmoids has been primarily surgical, but case reports of remission with chemotherapy or hormone therapy have been reported (10).

Head and neck desmoids frequently involve vital structures, and residual disease is frequent after resection. Faulkner et al, (9) identified surgical margin status as the only significant prognostic factor for disease recurrence in patients with pediatric desmoid tumor. Postoperative radiation therapy leads to excellent control for positive margins and is an important adjunct if the morbidity of recurrence would be high or the chances for salvage low (5).Therefore if a desmoids tumor is resectable with acceptable morbidity and cosmetic and functional outcome, surgery is the treatment of choice. If the operative risk or cosmetic or functional morbidity is too great, radiation therapy alone to total dose of 55 to 60 Gy is recommended (3).

Radiation therapy alone for gross disease leads to tumor control in 70% to 80% or more of the cases (10).

The a cellular matrix may persist as a mass even if the cells are killed. Reduction in tumor size after radiation therapy may not occur.

The use of chemotherapeutic and other systemic agents may be a reasonable alternative to avoid radiotherapy in the growing child, although chemotherapy also carries a risk for potentially adverse side effects, such as second malignancies, fertility problems, and cardio toxicity (11).

If the margins are positive after surgery there is an advantage for patients who received adjuvant chemotherapy compared with patients who did not receive adjuvant treatment (6).

## Conclusion

Primary surgery with negative margins is the treatment of choice for children with desmoids tumors. An MDT decision making and a team work is the cornerstone of success. In patients with unresectable tumors, the use of chemotherapy and/or radiotherapy in children with desmoids tumor may be a reasonable alternative.

## References

[1] ShieldsCJ, Winter DC, KirwanWO, Redmond HP (2001). Desmoid tumors. Eur J SurgOncol.

[2] Reitamo JJ, Scheinin TM, Hayry P (1986). The desmoid syndrome: mew aspects in the cause, pathogenesis and treatment ofthedesmoid tumor. Am J Surg.

[3] Timothy Anderson, Gregory S, Weinstein, John Harwick (2000). Hypopharyngealdesmoidtumor. Otolaryngology Head Neck Surg.

[4] Koeda S, Nagasaka H, Kumamoto H, Kawamura H (2005). Extra-abdominal fibromatosis of the cheek: report ofa case. J Oral Maxillofac Surg.

[5] Goy BW, Lee SP, Eilber F (1997). The role of adjuvant radiotherapyin the treatment of resectabledesmo idtumors. Int J RadiatOncolBiolPhys.

[6] Buitendijk S, van de Ven CP, Dumans TG (2005). Pediatric aggressive fibromatosis: a retrospective analysis of 13 patients and review of literature. Cancer.

[7] Wever de I, Cin dal P, Fletcher CDM, Mandahl N, Mertens F, Mitelman F (2000). Cytogenetic,clinical, and morphologic correlations in 78 cases of fibromatosis: a report from the CHAMP Study Group. Mod Pathol.

[8] Lackner H, Urban C, Kerbl R, Schwinger W (1997). Noncytotoxicdrug therapy in children with unresectabledesmoidstumors. Cancer.

[9] Faulkner LB, Hajdu SI, Kher U, Quaglia M La, Exelby P R, Heller G (1995). Pediatric desmoids tumor: retrospective analysis of 63 cases. J ClinOncol.

[10] Acker JC, Bossen EH, Halperin EC (1993). The management of desmoidtumors. Int J Radiat Oncol Biol Phys.

[11] Buitendijk S, Van de Ven CP, Dumans TG, den Hollander JC, Nowak PJ, Tissing WJ (2005). Pediatric aggressive fibromatosis: a retrospective analysis of 13 patients and review of literature. Cancer.

